# In vivo imaging with two-photon microscopy to assess the tumor-selective binding of an anti-CD137 switch antibody

**DOI:** 10.1038/s41598-022-08951-1

**Published:** 2022-03-22

**Authors:** Chisato Kaneko, Haruka Tsutsui, Kazuhisa Ozeki, Masaki Honda, Kenta Haraya, Yoshinori Narita, Mika Kamata-Sakurai, Junichi Kikuta, Mitsuyasu Tabo, Masaru Ishii

**Affiliations:** 1 Translational Research Division, Chugai Pharmaceutical Co., Ltd., 1-135, Komakado, Gotemba, Shizuoka 412-8513 Japan; 2 Research Division, Chugai Pharmaceutical Co., Ltd., 1-135, Komakado, Gotemba, Shizuoka 412-8513 Japan; 3 Chugai Pharmabody Research Pte. Ltd., 3 Biopolis Drive, #07-11 to 16, Synapse, Singapore, 138623 Singapore; 4 Research Division, Chugai Pharmaceutical Co., Ltd., 200, Kajiwara, Kamakura, Kanagawa 247-0570 Japan; 5 Department of Immunology and Cell Biology, Graduate School of Medicine and Frontier Biosciences, 2-2, Yamadaoka, Suita, Osaka 565-0871 Japan; 6 WPI-Immunology Frontier Research Center, Osaka University, 3-1, Yamadaoka, Suita, Osaka 565-0871 Japan; 7 Laboratory of Bioimaging and Drug Discovery, National Institutes of Biomedical Innovation, Health and Nutrition, 7-6-8, Saito-Asagi, Ibaraki, Osaka 567-0085 Japan

**Keywords:** Fluorescence imaging, Multiphoton microscopy, Cancer, Pharmacokinetics, Biologics

## Abstract

STA551, a novel anti-CD137 switch antibody, binds to CD137 in an extracellular ATP concentration-dependent manner. Although STA551 is assumed to show higher target binding in tumor tissues than in normal tissues, quantitative detection of the target binding of the switch antibody in vivo is technically challenging. In this study, we investigated the target binding of STA551 in vivo using intravital imaging with two-photon microscopy. Tumor-bearing human CD137 knock-in mice were intravenously administered fluorescently labeled antibodies. Flow cytometry analysis of antibody-binding cells and intravital imaging using two-photon microscopy were conducted. Higher CD137 expression in tumor than in spleen tissues was detected by flow cytometry analysis, and T cells and NK cells were the major CD137-expressing cells. In the intravital imaging experiment, conventional and switch anti-CD137 antibodies showed binding in tumors. However, in the spleen, the fluorescence of the switch antibody was much weaker than that of the conventional anti-CD137 antibody and comparable with that of the isotype control. In conclusion, we were able to assess switch antibody biodistribution in vivo through intravital imaging with two-photon microscopy. These results suggest that the tumor-selective binding of STA551 leads to a wide therapeutic window and potent antitumor efficacy without systemic immune activation.

## Introduction

CD137 is a costimulatory receptor, and stimulation of CD137 promotes T-cell survival, proliferation and effector function^1,2^. Several anti-CD137 agonist antibodies are being developed for the treatment of cancer^3^. In clinical trials, two monoclonal antibodies, urelumab (BMS-663513) and utomilumab (PF-05082566), have been administered to patients with tumors^3^. However, urelumab has been found to cause severe hepatotoxicity in phase I and II studies^4^. Utomilumab shows lower toxicity than urelumab but has less antitumor efficacy^5,6^. To overcome the issues facing conventional anti-CD137 antibodies, we generated an anti-CD137 agonist switch antibody, STA551^7^. STA551 only binds to CD137 in the presence of ATP; the binding is not detectable in the absence of ATP. A previous study reported that an anti-CD137 switch antibody showed antitumor efficacy in various tumor models without systemic immune activation. A conventional anti-CD137 antibody induced splenomegaly, lymphadenopathy, and activation of T cells in normal tissues. On the other hand, the anti-CD137 switch antibody did not induce these responses in normal tissues. The data suggest that STA551 is a novel antibody that shows CD137-agonistic activity selectively in tumors.

Antitumor efficacy data in various mouse models and ex vivo analysis data have suggested that STA551 binds selectively to CD137 in tumors, but quantitative detection of conventional and switch antibody biodistribution in tumor and normal tissues under physiological conditions has been a challenge^7^. ATP exists inside and outside the cell, and the intracellular ATP concentration is much higher than the extracellular ATP (exATP) concentration^8^. Additionally, exATP levels are different between tissues. ExATP in tumor interstitial fluid is reported to be approximately 100 μM, whereas plasma and normal tissues contain low exATP levels (10–100 nM)^8–10^. When animals are autopsied and tissues are sampled in ex vivo experiments, the ATP concentrations change based on the physiological conditions. ExATP and/or intracellular ATP might be degraded spontaneously or enzymatically, or intracellular ATP might be released from the cells during the process of ex vivo analysis. Thus, it is difficult to quantitatively detect STA551 binding to the target under physiological conditions using ex vivo analysis. Clarifying the minimal binding of STA551 in normal tissues in vivo would provide clear evidence of the reduced level of systemic immune activation mediated by STA551. Therefore, we aimed to find a working method for elucidating STA551 biodistribution in vivo.

Confocal fluorescence microscopy has been widely used to observe cell physiology^11^. This tool enables observation of high-resolution fluorescence images of cells and tissues. Since confocal microscopy uses single-photon absorption processes to produce images, it can only visualize tissues at depths of up to 100 μm^12^. In the last two decades, two-photon microscopy has been developed, which is an advanced form of microscopy that uses two-photon absorption processes to visualize images^13,14^. Since two-photon microscopy uses long-wavelength lasers that have low energy, it causes low phototoxicity and can be used for long-term imaging in a living animal. In addition, long-wavelength light can penetrate deeper into tissues than short-wavelength light, which enables observation not only of the surface but also of deep tissues. Another advantage of this imaging technology is its high resolution. Compared to Positron emission tomography (PET) and/or single-photon emission computed tomography (SPECT) technology, two-photon imaging technology gives higher-resolution images. High-resolution images enable observation of detailed aspects of cell physiology, such as cell morphology, cell movement, and cell–cell interactions^15,16^. Since two-photon microscopy is much better for intravital imaging than single-photon microscopy, two-photon technology has recently been utilized to detect cell physiology in several tissues, including bone, brain and tumor tissues^17–20^.

Intravital imaging using two-photon microscopy may be the most appropriate way to detect switch antibody binding to target cells for three reasons. First, two-photon microscopy can visualize various tissues, including tumor, spleen and lymph node tissues, to a considerable depth. The difference in distribution between tumor and normal tissues should be detected. Second, it enables images to be obtained in a living animal with physiological ATP concentrations in each tissue. Compared to ex vivo analysis, intravital imaging enables antibody binding to be evaluated without injuring the cells or significantly interfering with the ATP concentration. Thus, the binding of a switch antibody should be able to be detected under physiological conditions by intravital imaging. Third, high-resolution images enable observation of antibody binding to target cells.

In this study, we aimed to clarify the target binding of STA551 in tumor and normal tissues in vivo. First, we investigated CD137 expression in tumor-bearing human CD137 knock-in mice (hCD137-KI mice) and identified cell populations expressing CD137. Second, we administered a conventional CD137-agonistic antibody to tumor-bearing hCD137-KI mice and detected antibody-binding cells by flow cytometry. Finally, we administered the switch or conventional CD137-agonistic antibody to mice and confirmed the different distributions of these antibodies in the tumor and spleen using two-photon microscopy.

## Results

**Figure 1:**
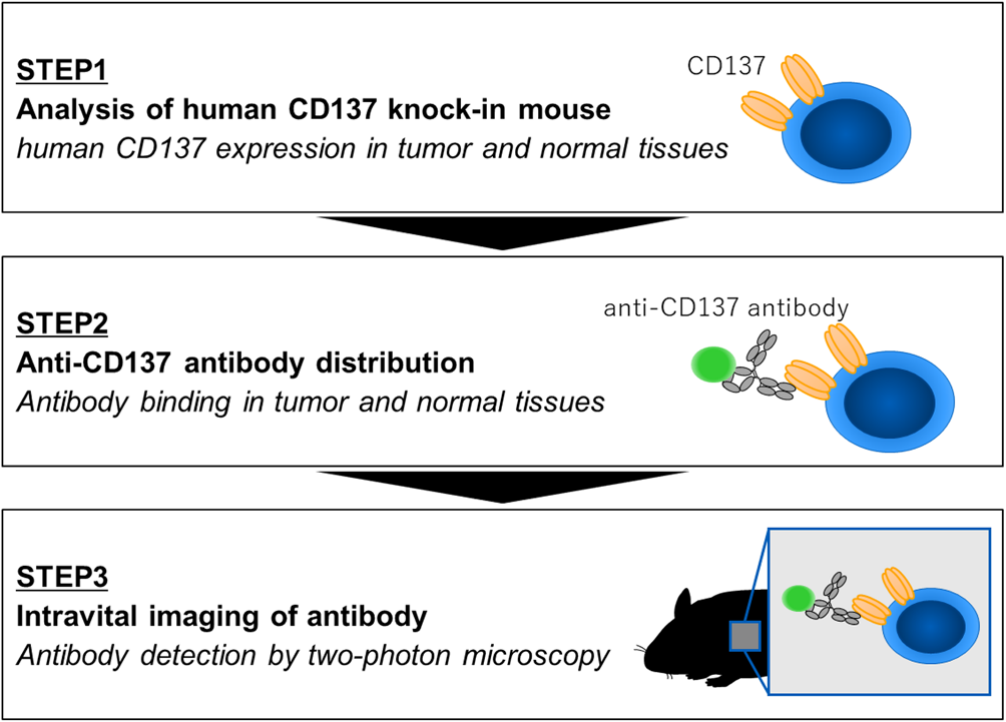
Research strategy for detection of antibody binding in tissues by two-photon microscopy. In Step 1, human CD137 expression was examined in tumor-bearing hCD137-KI mice. In Step 2, a fluorescently labeled anti-CD137 antibody was administered to hCD137-KI mice. Finally, antibody-binding cells in the tumor and spleen were detected by two-photon microscopy in Step 3.

To clarify the target binding of STA551 in tumor and normal tissues in vivo, we used a three-step process (Fig. 1). STA551 binds to CD137 in humans and cynomolgus monkeys but does not bind to murine CD137^7^. Therefore, we used hCD137-KI mice^7^ to investigate STA551 binding to the target.

**Figure 2:**
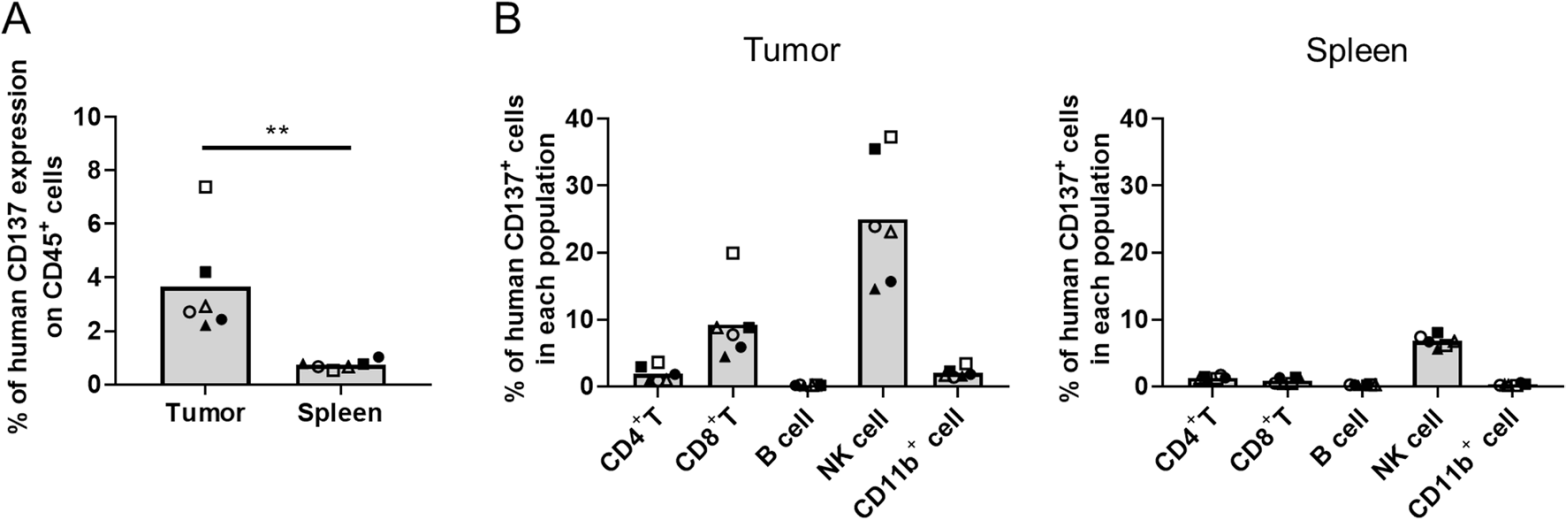
Human CD137 expression in LLC1/OVA/hGPC3-bearing hCD137-KI mice. Tumors and spleens were sampled at 24 h after administration of PBS (closed symbols) or an isotype control antibody (open symbols). (**A**) Human CD137 expression on CD45^+^ cells in tumor and spleen tissues. The individual values and mean for each tissue are shown. **P < 0.01 by t test. (**B**) Human CD137 expression on each type of cell. The percentage of human CD137-positive cells for each cell type was calculated by flow cytometry. The individual values and mean for each cell type are shown.

To investigate CD137 expression in hCD137-KI mice, we created an LLC1/OVA/hGPC3 model and detected CD137 expression by flow cytometry (Fig. 2). We first analyzed CD137 expression on CD45^+^ cells in the tumor and spleen. CD45^+^ cells in tumor tissue showed higher CD137 expression than those in spleen tissue (Fig. 2A). CD137 expression was observed in 2–8% of CD45^+^ cells in tumor tissue and in fewer than 2% of CD45^+^ cells in spleen tissue. Among CD45^+^ cells, CD8^+^ T cells and NK cells had high CD137 expression (Fig. 2B). CD137 expression on CD4^+^ T cells and CD11b^+^ cells was also detected (Fig. 2B). These data suggested that the anti-CD137 antibody distributed more to tumor tissue than to spleen tissue and bound to CD45^+^ cells, especially CD8^+^ cells and NK cells.

**Figure 3:**
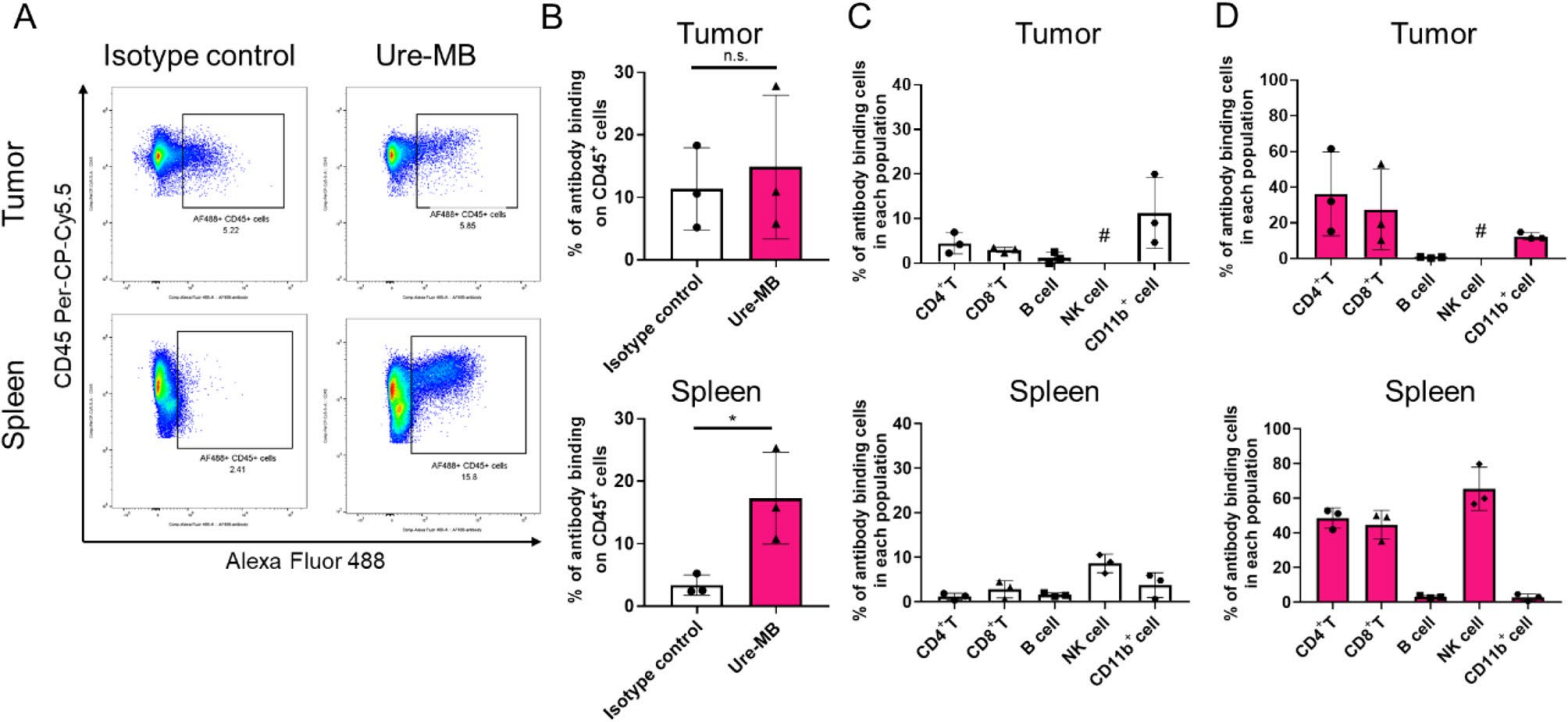
Anti-human CD137 antibody-binding cells in LLC1/OVA/hGPC3-bearing hCD137-KI mice. An Alexa Fluor 488-labeled isotype control antibody or Ure-MB was administered twice at a dose of 1 mg/kg. The tissues were sampled the day after the second administration. (**A**) Representative data plot of Alexa Fluor 488-labeled antibody-binding CD45^+^ cells in tissues. The population in the square represents Alexa Fluor 488-positive cells. (**B**) Percentage of Alexa Fluor 488-labeled antibody-binding CD45^+^ cells. Alexa Fluor 488-positive cells were detected in each tissue. The data were collected from three mice in each group. *p < 0.05 by t test. (**C,D**): (**C**) Alexa Fluor 488-labeled isotype control antibody binding and (**D**) Alexa Fluor 488-labeled Ure-MB binding for each type of cell in the tumor and spleen. The percentage of Alexa Fluor 488-positive cells for each cell type was calculated by flow cytometry. #NK cells in tumors were not detected in this experiment.

In previous research, to evaluate the in vivo antitumor efficacy of STA551, tumor-bearing mice were treated with Sta-MB and Ure-MB. Sta-MB has the same variable region as STA551 and has MB as the constant region, which is an engineered constant region of mouse IgG1 to increase binding activity to mouse Fc gamma receptor II (FcγRII). Ure-MB has a urelumab-like Fab and MB as the constant region. Ure-MB was used as a conventional CD137 agonist antibody, in contrast to Sta-MB, an anti-CD137 switch antibody^7^. To investigate anti-CD137 antibody binding in tissues, we administered Alexa Fluor 488-labeled Ure-MB and an isotype control antibody (anti-KLH-MB) to tumor-bearing hCD137-KI mice. These antibodies were administered at a dose of 1 mg/kg twice. Sta-MB was not used because it can potentially dissociate from the target during the process of ex vivo analysis. Ure-MB was detected not only in the tumors but also in the spleen tissues (Fig. 3A,B). Ure-MB bound to 5–30% of CD45^+^ cells in tumors and 10–25% of CD45^+^ cells in the spleen. In addition, Ure-MB bound to CD4^+^ T cells, CD8^+^ T cells and CD11b^+^ cells (Fig. 3D). NK cells in tumors were not detected in this experiment. In tumors, the isotype control antibody bound to CD45^+^ cells, especially CD11b^+^ cells (Fig. 3B,C). CD137 expression was not evaluated because Ure-MB binds to the same site of CD137 as the detection antibody. Given that the Ure-MB binding population was consistent with the CD137-expressing population shown in Fig. 2B, Ure-MB should bind to CD137-expressing immune cells. Taken together, these results indicate that Ure-MB binds to targets in both tumor and normal tissues.

**Figure 4:**
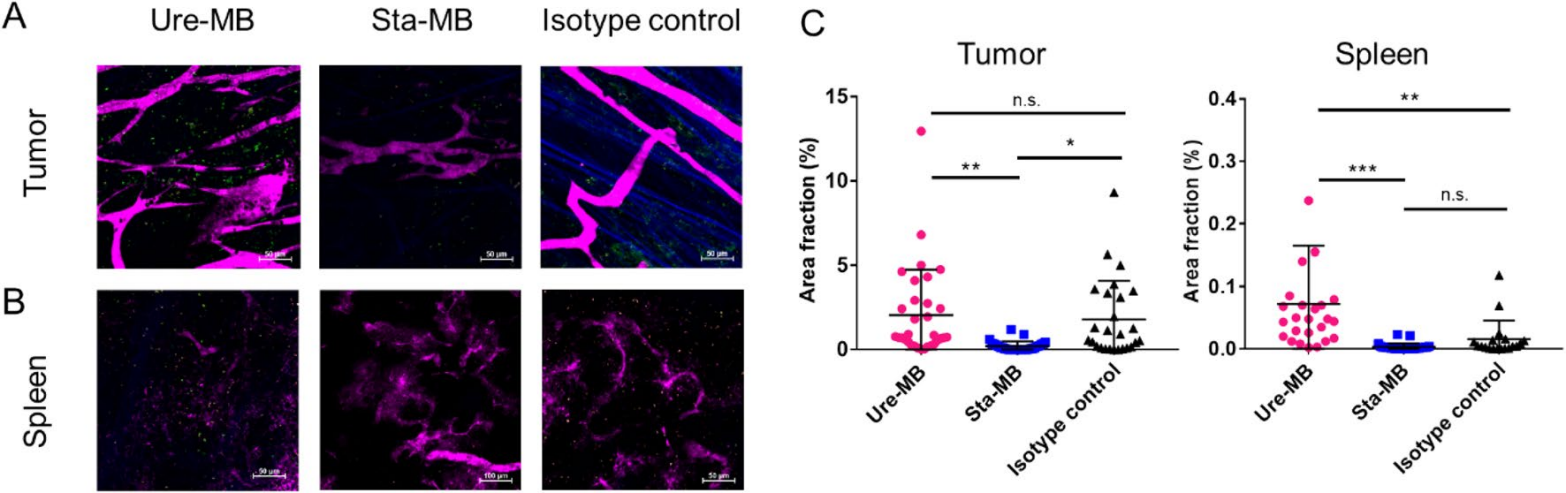
Detection of switch and nonswitch antibody-binding cells by two-photon microscopy. An Alexa Fluor 488-labeled isotype control antibody, Ure-MB or Sta-MB was administered twice at a dose of 1 mg/kg. Intravital imaging by two-photon microscopy was conducted the day after the second administration. (**A,B**) Representative images of Alexa Fluor 488-labeled antibody distribution in (**A**) tumor and (**B**) spleen tissues. To visualize blood vessels, Qtracker 655 vascular labels were intravenously administered just before observation by two-photon microscopy. Green, Alexa Fluor 488-labeled antibody; magenta, blood vessels; blue, collagen fibers. (**C**) Quantitative analysis of Alexa Fluor 488-labeled antibody fluorescence. The antibody-binding regions in the tumor and spleen were detected from images of three or four mice. **p < 0.01, ***p < 0.001 by Tukey’s multiple comparisons test.

To verify the target binding of STA551 in vivo, we investigated the distribution of Sta-MB in tumor and spleen tissues. Alexa Fluor 488-labeled Ure-MB, Sta-MB, and isotype control antibodies were administered to tumor-bearing hCD137-KI mice and detected by two-photon microscopy. In tumors, fluorescence from all antibodies was detected (Fig. 4A,C, Suppl Fig. 2). However, Sta-MB showed weaker fluorescence in the spleen than Ure-MB (Fig. 4B,C), and the Sta-MB fluorescence was comparable to that of the isotype control antibody. These data suggested that Sta-MB distributes and binds differently in the tumor and spleen in vivo and shows little binding to CD137 in the spleen.

## Discussion

In this study, we revealed that an anti-CD137 switch antibody binds to target cells differently in tumor and spleen tissues in vivo by using two-photon microscopy. We first examined the CD137 expression levels in tumor and spleen tissues in tumor-bearing hCD137-KI mice. A conventional anti-CD137 antibody was then administered to tumor-bearing hCD137-KI mice to determine the distribution of the antibody to tumor and spleen tissues. Finally, two-photon microscopy was used to detect the distribution of the anti-CD137 switch antibody to tumor and spleen tissues in vivo.

To confirm the different binding abilities of STA551 between tissues in vivo, we needed to detect the distribution of antibodies in a noninvasive way to minimize ATP concentration changes in tissues. Therefore, we used two-photon microscopy to detect the antibodies intravitally. In experiments using two-photon microscopy, it can be time-consuming to prepare animals, optimize imaging, and monitor the animals. On the other hand, in ex vivo analysis using flow cytometry, several experimental conditions, such as the dosing regimen, can be verified with improved throughput. In addition, ex vivo studies can detect the expression of target molecules and the binding of antibodies and identify the types of cells. Establishing optimal imaging conditions for intravital imaging by using ex vivo flow cytometric analysis would be useful for studying antibody distribution in vivo.

In this study, in vivo imaging revealed that Sta-MB distributed and bound to the cells in tumor tissue much more than in spleen tissue. It has been suggested that the toxicity induced by anti-CD137 antibodies is CD137 dependent and that T-cell and macrophage infiltration and cell-secreted cytokines are involved in the pathogenesis^21–23^. Ure-MB was highly distributed to not only tumor tissue but also spleen tissue, and it seemed to lead to systemic toxicity by inducing CD137 signaling in normal tissues. Our findings suggest that the tumor-selective target-binding ability of STA551 avoids the systemic reaction caused by conventional anti-CD137 antibodies.

Ure-MB, a conventional anti-CD137 antibody, bound to cells in tumor and spleen tissues. Ure-MB mainly bound to T cells and NK cells, consistent with cell populations in which CD137 expression was observed (Fig. 2B) or previously reported^24^. This suggested that Ure-MB bound to the cells in a CD137-dependent manner. There were two possible reasons why the binding of Ure-MB was greater than the expression of CD137. The first reason is that the administration of antibody increased the expression of CD137 molecules. CD137 agonist signals are known to activate T cells and other immune cells, leading to increased expression of CD137^25^. Ure-MB administered to mice might bind to CD137, introduce CD137 agonist signals in the tumor and spleen and induce increased expression of CD137 in tissues. The second reason is that the Fc region mediates binding of Ure-MB. Ure-MB, the isotype control antibody and Sta-MB have engineered Fc regions that bind to murine FcγRs, particularly FcγRII^7^, and FcγRII is predominantly expressed in myeloid lineage cells^26^. The isotype control antibody bound to CD45^+^ cells, especially CD11b^+^ cells, suggesting that the antibody bound to the cells via the Fc region. However, the binding of Ure-MB to CD11b^+^ cells was comparable with that of the isotype control antibody in either tumor or spleen tissue, suggesting that Fab-mediated binding was more prevalent than Fc-mediated binding. In addition, since the main Ure-MB-binding cells were confirmed to be CD137-expressing cells, such as T cells and NK cells, binding of Ure-MB to cells was thought to be primarily mediated by Fab.

There are some limitations to detecting antibodies in vivo by intravital imaging with two-photon microscopy. Two-photon microscopy can detect fluorescently labeled antibodies whether the antibodies are bound or unbound to the cells. In this study, the binding of the isotype control antibody observed in tumors seemed similar to that of Ure-MB, and Sta-MB showed poor binding. As shown in Table 1 and Suppl Fig. 1, the concentration of the isotype control antibody in tumors was higher than that of the other two antibodies, and Sta-MB had the lowest concentration among the three antibodies. Therefore, it is possible that more fluorescence derived from unbound isotype control antibodies, such as antibodies present in the interstitial fluid, and less fluorescence derived from unbound Sta-MB were detected in the intravital imaging experiment. Regarding the isotype control antibody, Fc-mediated binding was also detected because binding to CD11b^+^ cells was observed in flow cytometry analysis. In addition, only a limited area of tissues can be observed by two-photon microscopy because of the limited penetration of the laser into tissues and because of the manipulation of tissues. Since tissues, especially tumors, are not homogeneous, the extracellular ATP concentration is assumed to be heterogeneous within the tumor tissue. Hence, it is possible that Sta-MB, which binds to the target in the presence of a high extracellular ATP concentration, was observed at reduced levels in tumors for this reason. Another possible reason for the poor binding of Sta-MB in tumors is internalization of the antibody into the cell. Although a previous study has reported that antibody internalization does not differ considerably between Sta-MB and Ure-MB^7^, the exact internalization property in vivo is not known. Thus, it is possible that Sta-MB was quickly internalized into cells and degraded in this study. The fluorescence of Sta-MB detected in tumors was low in this study, while Sta-MB showed a stronger antitumor effect than Ure-MB in a previous efficacy study that used a similar dosing regimen to that in this research^7^. Therefore, Sta-MB is considered to bind CD137 and exhibit antitumor efficacy via CD137-agonistic activity in tumors.

**Table 1:** Antibody concentrations in plasma and tissues.

Antibody	Concentration (µg/mL plasma or µg/g tissue)			T/P	
	Plasma	Spleen	Tumor	Spleen	Tumor
Ure-MB	4.7	1.2	1.1	0.26	0.25
Sta-MB	5.5	0.5	0.6	0.09	0.11
Isotype control	8.0	1.5	5.0	0.19	0.62

An Alexa Fluor 488-labeled isotype control antibody, Ure-MB or Sta-MB was administered twice at a dose of 1 mg/kg. Tissues were collected the day after the second administration. The antibody concentrations in plasma and tissue lysates were measured by electrochemiluminescence (ECL) assay.

STA551 is designed to strongly bind to CD137 in the presence of 100 μM ATP but not in the absence of ATP^7^. Murine ATP levels have been reported to be approximately 100 μM in tumor tissues and 10–100 nM in normal tissues^8,9,27^. However, it is difficult to measure exact ATP concentrations under physiological conditions because ATP concentrations change depending on the sampling and measurement conditions due to degradation of ATP and release of intracellular ATP. This study revealed that Sta-MB showed binding in tumors and little binding in the spleen. These data suggest that under physiological conditions, tumors have ATP levels of 100 μM or higher, and normal tissues have lower ATP levels. The present imaging results may be useful for estimating ATP levels in tissues under physiological conditions. In addition, human ATP levels have been reported to be 10–100 nM in normal tissues^27^ and more than 10 μM in tumors; thus, there is an approximately 1000-fold difference in ATP concentration between tumor and normal tissues, similar to the situation in mice^28^. Considering the similarity in ATP distributions in human and mouse tissues, it is anticipated that STA551 will also exhibit tumor-selective binding in humans. Thus, STA551 is expected to exert antitumor efficacy with tumor-selective CD137 signals while reducing systemic reactions, even in human patients.

In conclusion, we showed that STA551 distributes to tumors but distributes little to the spleen. This STA551 distribution clarifies why STA551 works in tumors but not in normal tissues. Although intravital imaging with two-photon microscopy has some limitations in detecting antibodies, this technology is useful for noninvasive or intravital observation, such as detection of switch antibodies. Ex vivo techniques such as flow cytometry can be used in combination with two-photon microscopy to set experimental conditions and further interpret intravital imaging results. Because of its lower distribution in normal tissues than in tumor tissues, STA551 could be a promising therapeutic antibody for patients with cancers that are currently difficult to treat.

## Methods

### Cell line

LLC1/OVA/hGPC3 cells were established by transfecting human GPC3 and chicken ovalbumin (OVA)-expressing plasmids into LLC1 cells, which were purchased from the ATCC^7,29^.

### Animals

The animal studies were carried out in compliance with the ARRIVE guidelines (https://arriveguidelines.org/). All animal studies were conducted in accordance with the animal treatment policy of the Institutional Animal Care and Use Committee (IACUC) at Chugai Pharmaceutical Co., Ltd., and the Animal Experiments Committee of Osaka University. The animal experiments were performed in accordance with the Guidelines for the Care and Use of Laboratory Animals at Chugai Pharmaceutical Co., Ltd., which is accredited by the Association for Assessment and Accreditation of Laboratory Animal Care (AAALAC) International. All animal studies were approved by the Institutional Animal Care and Use Committee (IACUC) at Chugai Pharmaceutical Co., Ltd., and the Animal Experiments Committee of Osaka University. To create a subcutaneous tumor model for the in vivo antibody distribution study, 1.0 × 10^6^ LLC1/OVA/hGPC3 cells^7^ were subcutaneously inoculated into 8-week-old hCD137-KI male mice^7^. The hCD137-KI mice were generated by replacing mouse CD137 with human CD137. They did not express the mouse CD137 gene or protein but rather expressed the human CD137 gene and protein^7^.

### Antibody labeling

An Alexa Fluor 488 Antibody Labeling Kit (Thermo Fisher Scientific) was used to label Ure-MB, Sta-MB, and the isotype control antibody (anti-KLH-MB). The binding of labeled Ure-MB and Sta-MB to human CD137 in the presence and absence of ATP was determined by Biacore assay.

### In vivo study in hCD137-KI mice

The in vivo study was performed according to previously reported procedures^30^ with some modifications. The experimental conditions, including the dosing regimen, were set up to align with the conditions under which Sta-MB showed antitumor efficacy in a previous study^7^. Approximately 3 weeks after LLC1/OVA/hGPC3 tumor inoculation, 1 mg/kg Alexa Flour 488-labeled isotype control antibody or PBS was intravenously administered once, and CD137 expression was investigated. For the antibody distribution study, 1 mg/kg Alexa Flour 488-labeled antibodies were intravenously administered on Days 0 and 3. Mice were anesthetized with isoflurane at 24 h after the administration. The mice were then observed by intravital two-photon microscopy or their plasma and tissues were collected for single-cell analysis and determination of the antibody concentrations in tissues. To determine the concentrations in tissues, each tissue sample was homogenized with a TissueLyser II (Qiagen) in lysis buffer (Cell Signaling Technology #9803) supplemented with cOmplete™ Protease Inhibitor Cocktail (Roche #04693116001). The homogenate was centrifuged at 14,000 rpm for 15 min at 4 °C, and the supernatant was collected for analysis. The antibody concentrations in plasma and tissue lysate were measured by electrochemiluminescence (ECL) assay using MULTI-ARRAY Standard 384-well plates (Meso Scale Diagnostics) coated with recombinant human CD137 (Sino Biological) or keyhole limpet hemocyanin (KLH) from *Megathura crenulata* (Sigma) and a biotinylated anti-mouse IgG antibody (Southern Biotech). The ECL signals were detected with a MESO SECTOR S 600 (Meso Scale Diagnostics) after adding SULFO-TAG-labeled Streptavidin (Meso Scale Diagnostics).

### Flow cytometry

To analyze target expression and antibody distribution by flow cytometry, single-cell suspensions of each excised organ were prepared according to the manufacturer’s protocol using a tumor dissociation kit (Miltenyi Biotec) or dissociated using glass slides. After lysing the red blood cells with Pharm Lyse (BD Biosciences), the cell suspensions were reconstituted with PBS containing 0.5% BSA and 2 mM EDTA to stain the cell surface and subcellular epitopes. The cell suspensions were stained with anti-CD45 Per-CP-Cy5.5 (30-F11), anti-CD3e BUV496 (145-2C11), anti-CD4 BV786 (RM4–5), anti-CD8a PE-Cy7 (53–6.7), anti-CD19APC (1D3), anti-CD11b APC-Cy7 (M1/70), anti-CD49b BV421 (DX5), and anti-human CD137 PE (4B4-1) purchased from BD Biosciences. The cell suspensions were also stained with Zombie Aqua (BioLegend). Following fixation, data were acquired on a FACS LSRFortessa™ X-20 (BD Biosciences). Data analysis was performed using FlowJo software (version 10.2).

### Intravital two-photon microscopy imaging

As a pretreatment before intravital two-photon microscopy imaging, mice were shaved, and the hair was removed with depilatory cream to prevent it from entering the visual field. The spleen and tumor were then surgically exposed and covered with cover glass using n-butyl cyanoacrylate glue (Vetbond Tissue Adhesive, 3M). Qtracker 655 Vascular Labels (Thermo Fisher Scientific) were intravenously administered into the mice just before imaging of the spleen and tumor to visualize the blood vessels in these tissues. An inverted multiphoton microscope (A1R-MP, Nikon) equipped with multi-immersion objectives (20×, Plan Fluor, numerical aperture [NA], 0.75, Nikon) was used to observe the tumor and spleen. The microscope was driven by a Chameleon Vision II Ti:Sapphire laser (Coherent) tuned to 930 nm. The fluorescence was detected by an external non-descanned detector with four channels (Nikon), three dichroic mirrors (495, 560, and 593 nm) and four bandpass filters: 492 nm for the second harmonic generation (SHG) signal, 525/50 nm for Alexa Fluor 488, 575/25 nm for tdTomato, and 629/56 nm for Qtracker 655. Four to 11 images of approximately 300 μm × 300 μm with a vertical step size of approximately 3 μm to a depth of approximately 100 μm were collected for each tissue and then analyzed by NIS-Elements integrated software (Nikon) to create the maximum intensity projection (MIP) images with median filters for noise reduction. The areas of the antibody-binding regions in the tumor and spleen were detected by ImageJ software. The antibody-binding regions were extracted from the MIP images. The area fractions were calculated as the fluorescence area/total area in all images.

### Statistical analysis

Statistical analyses were performed with GraphPad Prism 7.0 (GraphPad Software). The CD137 expression level and antibody distribution were compared by using Student’s t test. Antibody fluorescence in the two groups was compared using Tukey’s multiple comparisons test. Significant values are marked as *p < 0.05, **p < 0.01, ***p < 0.001, and n.s.

## Supplementary Information

Supplementary Figures.
