# Supplementary material for: In vivo imaging with two-photon microscopy to assess the tumor-selective binding of an anti-CD137 switch antibody
Source: Sci Rep. 2022 Mar 22;12:4907. doi: 10.1038/s41598-022-08951-1 (PMC8941111; doi:10.1038/s41598-022-08951-1)
Supplement: Supplementary file 1 — Supplementary Figures. [file 41598_2022_8951_MOESM1_ESM.pdf]

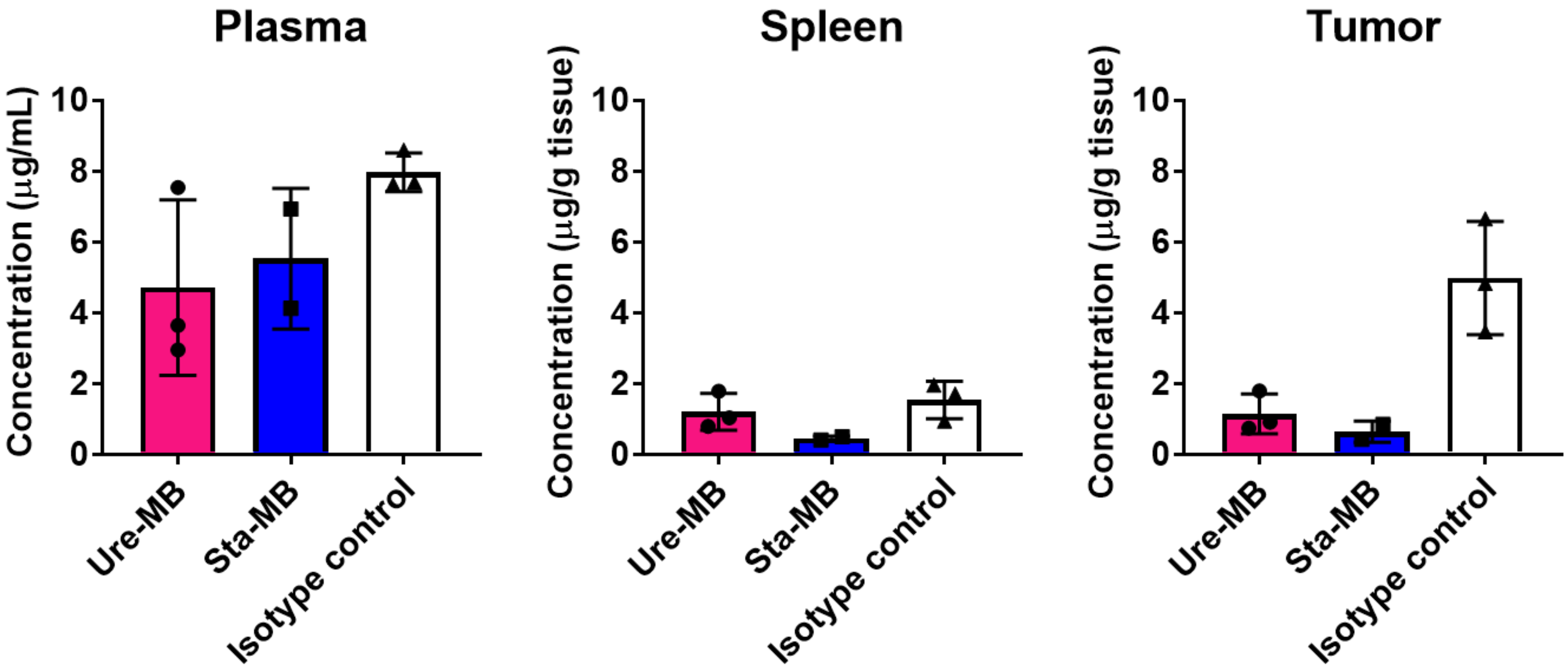

Supplemental Figure 1. Antibody concentration in plasma and tissues. Alexa Fluor 488-labeled isotype control antibody, Ure-MB or Sta-MB were administered twice at doses of 1 mg/kg. Blood and tissue samples were collected the day after the second administration. Antibody concentrations in plasma and tissue lysates were measured by an electrochemiluminescence (ECL) assay.

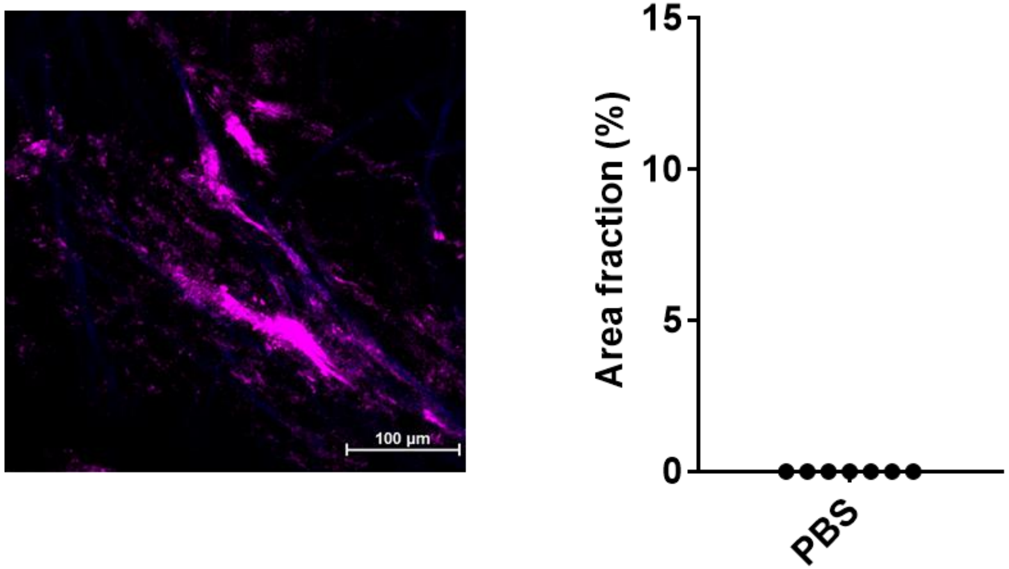

Supplemental Figure 2. Alexa Fluor 488 florescence was not detected in tumor of PBS administered mice by two-photon microscopy. Intravital imaging by two-photon microscopy was conducted 24 hours after the administration. To visualize blood vessels, Qtracker 655 vascular labels were intravenously administered just before observation of two-photon microscopy. Magenta, blood vessels; blue, collagen fiber. Area fraction of Alexa Fluor 488 florescence was calculated.
